# Cerebral Small Vessel Disease Burden Is Associated with Motor Performance of Lower and Upper Extremities in Community-Dwelling Populations

**DOI:** 10.3389/fnagi.2017.00313

**Published:** 2017-09-27

**Authors:** Ning Su, Fei-Fei Zhai, Li-Xin Zhou, Jun Ni, Ming Yao, Ming-Li Li, Zheng-Yu Jin, Gao-Lang Gong, Shu-Yang Zhang, Li-Ying Cui, Feng Tian, Yi-Cheng Zhu

**Affiliations:** ^1^Department of Neurology, Peking Union Medical College Hospital, Chinese Academy of Medical Sciences and Peking Union Medical College, Beijing, China; ^2^Department of Radiology, Peking Union Medical College Hospital, Chinese Academy of Medical Sciences and Peking Union Medical College, Beijing, China; ^3^State Key Laboratory of Cognitive Neuroscience and Learning and IDG/McGovern Institute for Brain Research, Beijing Normal University, Beijing, China; ^4^Department of Cardiology, Peking Union Medical College Hospital, Chinese Academy of Medical Sciences and Peking Union Medical College, Beijing, China; ^5^State Key Laboratory of Computer Science, Institute of Software, Chinese Academy of Sciences, Beijing, China

**Keywords:** cerebral small vessel disease (CSVD), burden, white matter hyperintensities (WMH), brain atrophy, motor performance, upper extremities, lower extremities

## Abstract

**Objective:** To investigate the correlation between cerebral small vessel disease (CSVD) burden and motor performance of lower and upper extremities in community-dwelling populations.

**Methods:** We performed a cross-sectional analysis on 770 participants enrolled in the Shunyi study, which is a population-based cohort study. CSVD burden, including white matter hyperintensities (WMH), lacunes, cerebral microbleeds (CMBs), perivascular spaces (PVS), and brain atrophy were measured using 3T magnetic resonance imaging. All participants underwent quantitative motor assessment of lower and upper extremities, which included 3-m walking speed, 5-repeat chair-stand time, 10-repeat pronation–supination time, and 10-repeat finger-tapping time. Data on demographic characteristics, vascular risk factors, and cognitive functions were collected. General linear model analysis was performed to identify potential correlations between motor performance measures and imaging markers of CSVD after controlling for confounding factors.

**Results:** For motor performance of the lower extremities, WMH was negatively associated with gait speed (standardized β = -0.092, *p* = 0.022) and positively associated with chair-stand time (standardized β = 0.153, *p* < 0.0001, surviving FDR correction). For motor performance of the upper extremities, pronation–supination time was positively associated with WMH (standardized β = 0.155, *p* < 0.0001, surviving FDR correction) and negatively with brain parenchymal fraction (BPF; standardized β = -0.125, *p* = 0.011, surviving FDR correction). Only BPF was found to be negatively associated with finger-tapping time (standardized β = -0.123, *p* = 0.012). However, lacunes, CMBs, or PVS were not found to be associated with motor performance of lower or upper extremities in multivariable analysis.

**Conclusion:** Our findings suggest that cerebral microstructural changes related to CSVD may affect motor performance of both lower and upper extremities. WMH and brain atrophy are most strongly associated with motor function deterioration in community-dwelling populations.

## Introduction

Motor impairments become more prevalent with advancing age (Lord et al., 2016). Cerebral small vessel disease (CSVD), which is characterized by neuroimaging changes, including white matter hyperintensities (WMH), lacunes, cerebral microbleeds (CMBs), perivascular spaces (PVS), and brain atrophy, may accelerate age-related motor deterioration (Wardlaw et al., 2013). Previous studies have demonstrated that WMH (Rosano et al., 2006, 2010; Baezner et al., 2008; Silbert et al., 2008; Blahak et al., 2009; Soumare et al., 2009; de Laat et al., 2010; Smith et al., 2015; Pinter et al., 2017), lacunar infarcts (de Laat et al., 2010; Smith et al., 2015), and CMBs (Pinter et al., 2017) are associated with slower gait speed. However, other cross-sectional and longitudinal analyses have suggested that lacunes, CMBs, and PVS are not related to gait disturbance (Soumare et al., 2009; de Laat et al., 2011; Pinter et al., 2017). Thus, the contribution of CSVD to motor performance of lower extremities has been widely accepted, while the importance of each imaging marker remains controversial.

Due to their lower impact on daily activities, upper extremity functions have been under-investigated when compared to walking impairment. Limited evidence indicates that deficits in hand motor functions in the aging population are related to deficits in activities of daily life, increased functional dependence, and even mortality (Scherder et al., 2008). In view of the coexisting cortical and subcortical lesions in CSVD, one can easily presume that there may also be deterioration of hand functions in elderly individuals with magnetic resonance imaging (MRI) markers of CSVD. However, motor performance of the upper extremities has not yet been well-studied in relation to CSVD burden.

In the current study, we quantitatively evaluated motor performance of lower and upper extremities in a community-dwelling cohort. We aimed to investigate the contribution of CSVD burden to motor changes in the lower and upper extremities, and to provide new insights on the importance of prevention and interventions for motor impairment in elderly individuals with CSVD burden.

## Materials and Methods

### Study Participants and Clinical Data Collection

The Shunyi study is a population-based prospective cohort study that was designed to investigate the risk factors and consequences of brain changes in community-dwelling adults in a Chinese population. All inhabitants aged 35 years or older and independently living from five villages of Shunyi, a suburb district of Beijing, were invited to participate this cohort study. From June 2013 to April 2016, a total of 1,787 participants agreed to join and accomplish standard baseline assessments, which included structured questionnaires, physical examination, and laboratory tests. All participants were invited to have brain MRI examination. Among those, 464 participates refused or had contradictions of MRI scanning, leaving 1,323 participants with brain MRI scans. In 2014, the participants were invited to have motor evaluations. From the 909 participants who had undergone both tests of brain MRI examination and motor evaluation, we excluded 58 with prior stroke, 14 with muscle strength levels lower than grade 3, and those with involuntary movements. Participants with poor MRI quality (*n* = 67) were also excluded, leaving 770 participants in the present analysis. The baseline characteristics of participants included and not included in the current study were balanced, except that the participants enrolled had a lower proportion of male and current smokers, and higher proportion of hyperlipidemia. All participants signed an informed consent form. The Medical Review Ethics Committee of Peking Union Medical College Hospital approved the study (reference number: B-160).

Cardiovascular risk factors were defined as follows: hypertension was defined as blood pressure ≥140/90 mmHg; diabetes mellitus (DM) was defined as a fasting plasma glucose level ≥7.0 mmol/L or 2-h plasma glucose level ≥11.1 mmol/L during an oral glucose tolerance test; hyperlipidemia was defined as total cholesterol >5.2 mmol/L or low-density lipoprotein >2.58 mmol/L; and current smoker was defined as an individual smoking at least 1 cigarette per day for more than 6 months before enrollment. Neurological examinations were performed at all sites. Cognitive status was evaluated by neurologists using the Mini Mental State Examination (MMSE), which is a test with total score ranging from 0 to 30.

### Assessment of Motor Performance

All participants underwent motor assessment of the lower and upper extremities. Motor parameters in this study included 3-m walking speed, time to perform 5-repeat chair-stand, 10-repeat pronation–supination, and 10-repeat finger-tapping. See Supplementary Materials for more detailed information.

### MRI Acquisition and Definitions of Imaging Markers and Severity

MRI acquisition was performed from July 2014 to April 2016 using a single 3-Tesla Siemens Skyra scanner (Siemens, Erlangen, Germany). Three-dimensional T1-weighted images were acquired using magnetization-prepared rapid gradient-echo sequences in the sagittal plane [repetition time (TR) = 2,530 ms; echo time (TE) = 3.43 ms; inversion time = 1,100 ms; field of view (FOV) = 256 mm × 256 mm; voxel size = 1 mm × 1 mm × 1.3 mm; flip angle = 8°; 144 sagittal slices]. T2-weighted images (TR = 6,000 ms; TE = 125 ms; FOV = 230 mm × 230 mm; flip angle = 90°; slice thickness = 5 mm, gap = 1 mm; 80 axial slices), fluid-attenuated inversion recovery (FLAIR) images (TR = 8,500 ms; TE = 81 ms; FOV = 230 mm × 230 mm; flip angle = 150°; slice thickness = 5 mm, gap = 1 mm; 80 axial slices), and susceptibility weighted imaging (SWI) images (TR = 27 ms; TE = 20 ms; FOV = 208 mm × 230 mm, flip angle: 15°; slice thickness = 1.5 mm; 80 axial slices) were acquired in the axial plane.

White matter hyperintensities on FLAIR scans were not hypointense or were only faintly hypointense on T1-weighted images. Periventricular white matter hyperintensities (PVWMH) and deep white matter hyperintensities (DWMH) were scored on axial FLAIR images using the Fazekas scale (Fazekas et al., 1987). Individuals with severe total WMH were defined as those with either PVWMH or DWMH rated as higher than 2 on the Fazekas scale.

Lacunes were defined as focal lesions ranging from 3 to 15 mm in size with the same signal characteristics as cerebrospinal fluid (CSF) on all sequences situated in basal ganglia (BG) or white matter (WM). Lacunar infarcts were rated on three-dimensional T1-weighted images, and T2 and FLAIR images were used to confirm the presence of lesions.

Cerebral microbleeds were defined using standard published criteria (Greenberg et al., 2009) as round or ovoid hypointense lesions on SWI sequences.

Dilated PVS were defined as lesions with CSF-like signals and round, ovoid, or linear shapes. These lesions had smooth delineated contours and were located in areas supplied by perforating arteries. Severities of PVS in areas of WM and BG were assessed using previously published methods (Zhu et al., 2010) and high-resolution three-dimensional T1-weighted images. T2-weighted images were used to confirm the presence of the lesions. Dilated PVS in WM (PVS-WM) were scored as follows: degree 1, <10 PVS in the total WM volume; degree 2, >10 PVS in the total WM volume and <10 PVS in the slice containing the greatest number of PVS; degree 3, 10–20 PVS in the slice containing the greatest number of PVS; and degree 4, >20 PVS in the slice containing the greatest number of PVS. Dilated PVS in BG (PVS-BG) were scored as follows: degree 1, <5 PVS; degree 2, 5–10 PVS; degree 3, >10 PVS, but still numerable; and degree 4, innumerable PVS. Severe PVS-WM or PVS-BG was defined as degrees 2 and 3 in the WM and BG, respectively.

Automated segmentation of the T1 images was conducted to obtain gray matter (GM), WM, and CSF probability maps. Total brain volume was then calculated as the sum of the total GM, total WM, and CSF volumes. The brain parenchymal fraction (BPF) was defined as the ratio of brain tissue volume (GM and WM volume) to intracranial volume.

Well-trained readers who were blinded to all clinical data rated WMH, lacunes, CMBs, and PVS independently. Intra-rater agreement was assessed in a random sample of 50 individuals with an interval of longer than 1 month between the first and second readings. Kappa values for the intra-rater agreements were 0.84 for PVWMH, 0.89 for DWMH, 0.73 for lacunes, 0.90 for CMBs, 0.71 for PVS-BG, and 0.61 for PVS-WM.

### Statistical Analysis

Continuous variables are expressed as mean and standardized deviation, and categorical variables are expressed as frequencies and proportions. Analyses of differences in motor performance based on different types of CSVD burden (severity of WMH, presence or absence of lacunes/CMBs, or severity of PVS-WM/PVS-BG) were carried out using Student’s *t*-tests or Wilcoxon–Mann–Whitney tests (Supplementary Material). Spearman’s correlation coefficients were used to assess the associations between motor parameters and BPF (Supplementary Material). The relationships between CSVD imaging markers and motor performance were evaluated using general linear model (GLM) analyses with each CSVD marker (total WMH, PVWMH, DWMH, lacunes, CMBs, PVS-WM, PVS-BG, and BPF) as a determinant and motor parameter (gait speed, time of chair-stand, time of pronation–supination, and time of finger-tapping) as outcome variables. The models used to assess motor performance of the lower extremities were adjusted as follows: Model 1 was adjusted for age, sex, and height; Model 2 was the same as Model 1, with further adjustments for MMSE scores and education (high school and above vs. below); and Model 3 was the same as Model 2, with adjustments for vascular risk factors (hypertension, DM, hyperlipidemia, and smoking status). The models used to assess motor performance of the upper extremities were adjusted as follows: Model 1 was adjusted for age and sex; Model 2 was the same as Model 1, with adjustments for MMSE and education (high school and above vs. below); and Model 3 was the same as Model 2, with adjustments for vascular risk factors (hypertension, DM, hyperlipidemia, and smoking status). For the regression analyses a false discovery rate (FDR) correction was applied to correct for multiple comparisons (Glickman et al., 2014). Statistical significance was defined as *p*< 0.05. Statistical analyses were conducted using SAS version 9.4 (SAS Institute, Inc., Cary, NC, United States).

## Results

### Demographic, Imaging, and Motor Characteristics

The participants’ demographics, vascular risk factor variables, imaging markers of CSVD burden, and motor performance are shown in **Table 1**. Of the 770 patients included in the final analysis, 269 (34.9%) were male, and the mean age of the participants was 57.2 years. Severe total WMH was observed in 159 (20.6%) subjects, of whom 146 (19.0%) had Fazekas scores of 2 or 3 for PVWMH. Ninety-two of these participants (11.9%) had Fazekas scores of 2 or 3 for DWMH. Lacunes, CMBs, severe PVS-WM, and severe PVS-BG were found in 117 (15.2%), 78 (10.1%), 125 (16.2%), and 112 (14.6%) participants, respectively. The mean 3-m walking speed was 0.9 m/s and the average time for the 5-repeat chair-stand was 8.9 s. The average times for 10-repeat pronation–supination and 10-repeat finger-tapping were 7.5 and 5.4 s, respectively (**Table 1**). Univariate analysis indicated that motor performance of the lower and upper extremities significantly differed between the different types of CSVD burdens, except for the different PVS-WM groups (Supplementary Table 1). Spearman’s correlation analyses also indicated that motor performances in the lower extremities and upper extremities were associated with BPF, which is a marker of brain atrophy (Supplementary Figure 1).

**Table 1:** Baseline characteristics of the study population.

Characteristics	Mean or percentage (*n* = 770)
Demographic and clinical characteristics	
Age (years)	57.2 (9.3)
Male	269 (34.9%)
Education	
Under high school	654 (85.2%)
High school and above	114 (11.8%)
Hypertension	387 (50.3%)
DM	129 (16.8%)
Hyperlipidemia	371 (48.2%)
Current smoker	165 (21.9%)
Height (cm)	159 (7.8)
MMSE	26.3 (3.6)
Neuroimaging characteristics	
Total WMH	159 (20.6%)
Mild	611 (79.4%)
Severe	159 (20.6%)
PVWMH	
Mild	624 (81.0%)
Severe	146 (19.0%)
DWMH	
Mild	678 (88.1%)
Severe	92 (11.9%)
Presence of Lacunes	117 (15.2%)
Presence of CMBs	78 (10.1%)
PVS-WM	
Degree 1 or 2	644 (83.8%)
Degree 3 or 4	125 (16.2%)
PVS-BG	
Degree 1 or 2	657 (85.4%)
Degree 3 or 4	112 (14.6%)
Total brain volume (mL)	1403 (124)
BPF	76.4 (3.1)
Motor performance characteristics	
Gait speed (m/s)	0.9 (0.3)
Chair-stand (s)	8.9 (2.1)
Pronation–supination (s)	7.5 (1.7)
Finger-tapping (s)	5.4 (1.8)


*DM, diabetes mellitus; MMSE, Mini Mental State Examination; WMH, white matter hyperintensities; PVWMH, periventricular white matter hyperintensities; DWMH, deep white matter hyperintensities; CMBs, cerebral microbleeds; PVS-WM, dilated perivascular spaces in white matter (WM); PVS-BG, dilated perivascular spaces in basal ganglia (BG); BPF, brain parenchymal fraction. Data are presented as mean (standard deviation) or *n* (%).*

### Associations between Imaging Markers of CSVD and Motor Performance of Lower Extremities

We used GLM to assess whether CSVD imaging markers were independently associated with motor performance of lower extremities, as assessed using gait speed and time of chair-stand (**Table 2**). After adjustments for age, sex, and height, total WMH was significantly associated with gait speed (standardized β = -0.106, *p* = 0.007). Additional adjustment for cognitive status and vascular risk factors minimally attenuated the association (standardized β = -0.092, *p* = 0.022). Similarly, PVWMH was significantly associated with gait speed after the adjustments (standardized β = -0.080, *p* = 0.047), while DWMH was not associated with this measure. However, the correlation between WMH and gait speed did not survive the FDR corrections (**Table 2**).

**Table 2:** Associations between imaging markers of CSVD and motor function of lower extremities.

CSVD imaging markers	Gait speed		Chair-stand	


	β (SE)	*p*	β (SE)	*p*
Total WMH				
Model 1	-0.106 (0.040)	0.007*	0.157 (0.038)	<0.0001**
Model 2	-0.098 (0.040)	0.014*	0.150 (0.038)	0.0001**
Model 3	-0.092 (0.040)	0.022*	0.153 (0.039)	<0.0001**
PVWMH				
Model 1	-0.096 (0.040)	0.016*	0.183 (0.038)	<0.0001**
Model 2	-0.087 (0.040)	0.029*	0.176 (0.038)	<0.0001**
Model 3	-0.080 (0.040)	0.047*	0.180 (0.039)	<0.0001**
DWMH				
Model 1	-0.076 (0.038)	0.045*	0.102 (0.037)	0.006**
Model 2	-0.069 (0.038)	0.069	0.100 (0.037)	0.007**
Model 3	-0.066 (0.038)	0.083	0.101 (0.037)	0.006**
Lacunes				
Model 1	-0.061 (0.038)	0.110	0.061 (0.037)	0.101
Model 2	-0.058 (0.038)	0.130	0.062 (0.037)	0.096
Model 3	-0.047 (0.039)	0.229	0.067 (0.038)	0.080
CMBs				
Model 1	0.008 (0.036)	0.826	0.057 (0.036)	0.107
Model 2	0.006 (0.037)	0.869	0.070 (0.036)	0.052
Model 3	0.009 (0.037)	0.806	0.069 (0.036)	0.058
PVS-WM				
Model 1	-0.026 (0.037)	0.477	0.005 (0.036)	0.896
Model 2	-0.029 (0.036)	0.420	0.004 (0.036)	0.901
Model 3	-0.027 (0.037)	0.457	0.007 (0.036)	0.853
PVS-BG				
Model 1	-0.041 (0.036)	0.257	-0.018 (0.036)	0.605
Model 2	-0.044 (0.036)	0.228	-0.020 (0.036)	0.582
Model 3	-0.049 (0.037)	0.182	-0.022 (0.036)	0.549
BPF				
Model 1	0.065 (0.051)	0.204	-0.081 (0.050)	0.108
Model 2	0.056 (0.051)	0.273	-0.068 (0.050)	0.171
Model 3	0.061 (0.052)	0.238	-0.067 (0.050)	0.186


*β, standardized β coefficient; CSVD, cerebral small vessel disease; WMH, white matter hyperintensities; CMBs, cerebral microbleeds; PVS-WM, dilated perivascular spaces in white matter; PVS-BG, dilated perivascular spaces in BG; BPF, brain parenchymal fraction. Model 1: adjustment for age, sex, and height. Model 2: additional adjustment for MMSE, education level (high school and above vs. below). Model 3: additional adjustment for vascular risk factors (hypertension, DM, hyperlipidemia, and smoking status). ^∗^Uncorrected *p* < 0.05.^∗∗^Surviving a false discovery rate (FDR)-corrected threshold of *p*(FDR) = 0.05.*

Total WMH, PVWMH, and DWMH were independently associated with time of chair-stand after adjustment for age, sex, and height (standardized β = 0.157, *p* < 0.0001; standardized β = 0.183, *p* < 0.0001; standardized β = 0.102, *p* = 0.006, respectively). Further adjustments and the FDR corrections did not change the findings (**Table 2**).

None of the other imaging markers of CSVD, including lacunes, CMBs, PVS-WM, PVS-BG, and BPF, was associated with gait speed or time of chair-stand (**Table 2**).

### Associations between Imaging Markers of CSVD and Motor Performance of Upper Extremities

General linear model was used to assess correlations between imaging markers of CSVD and motor performance of upper extremities, as assessed using time of pronation–supination and finger-tapping. In Model 1, which was adjusted for age and sex, increased time required to complete pronation–supination was associated with higher total WMH burden (standardized β = 0.173, *p* < 0.0001), PVWMH (standardized β = 0.180, *p* < 0.0001), and DWMH (standardized β = 0.115, *p* = 0.002), as well as with decreased BPF (standardized β = -0.141, *p* = 0.004). Further adjustments for cognitive status and vascular risk factors did not change the results, and the results survived the FDR corrections (**Table 3**).

**Table 3:** Associations between imaging markers of CSVD and motor function of upper extremities.

CSVD imaging markers	Pronation–supination		Finger-tapping	


	β (SE)	*p*	β (SE)	*p*
Total WMH				
Model 1	0.173 (0.038)	<0.0001**	0.018 (0.038)	0.628
Model 2	0.154 (0.038)	<0.0001**	0.008 (0.038)	0.833
Model 3	0.155 (0.038)	<0.0001**	0.007 (0.039)	0.846
PVWMH				
Model 1	0.180 (0.038)	<0.0001**	0.028 (0.038)	0.457
Model 2	0.160 (0.038)	<0.0001**	0.017 (0.038)	0.649
Model 3	0.163 (0.038)	<0.0001**	0.020 (0.039)	0.608
DWMH				
Model 1	0.115 (0.037)	0.002**	0.039 (0.036)	0.280
Model 2	0.106 (0.036)	0.003**	0.032 (0.036)	0.376
Model 3	0.104 (0.036)	0.005**	0.029 (0.037)	0.430
Lacunes				
Model 1	0.035 (0.037)	0.352	0.002 (0.037)	0.962
Model 2	0.035 (0.036)	0.341	0.001 (0.036)	0.984
Model 3	0.031 (0.038)	0.420	0.004 (0.038)	0.926
CMBs				
Model 1	0.059 (0.036)	0.102	0.034 (0.035)	0.335
Model 2	0.066 (0.036)	0.065	0.025 (0.036)	0.485
Model 3	0.065 (0.036)	0.071	0.026 (0.036)	0.479
PVS-WM				
Model 1	-0.014 (0.036)	0.692	-0.028 (0.035)	0.430
Model 2	-0.014 (0.035)	0.692	-0.024 (0.035)	0.490
Model 3	-0.023 (0.035)	0.511	-0.018 (0.035)	0.611
PVS-BG				
Model 1	0.030 (0.036)	0.395	-0.013 (0.035)	0.708
Model 2	0.026 (0.035)	0.456	-0.012 (0.035)	0.742
Model 3	0.022 (0.036)	0.535	-0.017 (0.036)	0.627
BPF				
Model 1	-0.141 (0.049)	0.004**	-0.120 (0.048)	0.013*
Model 2	-0.126 (0.048)	0.009**	-0.117 (0.048)	0.015*
Model 3	-0.125 (0.049)	0.011**	-0.123 (0.049)	0.012*

*β, standardized β coefficient; CSVD, cerebral small vessel disease; WMH, white matter hyperintensities; CMBs, cerebral microbleeds; PVS-WM, dilated perivascular spaces in WM; PVS-BG, dilated perivascular spaces in BG; BPF, brain parenchymal fraction. Model 1: adjustment for age and sex. Model 2: additional adjustment for MMSE, education level (high school and above vs. below). Model 3: additional adjustment for vascular risk factors (hypertension, DM, hyperlipidemia, and smoking status). ^∗^Uncorrected *p* < 0.05. ^∗∗^Surviving a FDR-corrected threshold of *p*(FDR) = 0.05.*

In Model 1, which was adjusted for age and sex, only BPF was found to be significantly associated with finger-tapping time (standardized β = -0.120, *p* = 0.013). When the model was further adjusted for cognitive status and vascular risk factors, the association did not change. However, the correlation between BPF and finger-tapping did not survive the FDR correction. No significant associations between performance on the finger-tapping task and the CSVD imaging markers of WMH, lacunes, CMBs, PVS-WM, and PVS-BG were found (**Table 3**).

## Discussion

In this population-based cohort study, we found that higher WMH burden was significantly associated with motor deficits in the lower and upper extremities. In contrast, brain atrophy contributed only to decreased speed of upper extremities performance. No associations were found between lacunes, CMBs, and PVS, and motor parameters of the lower or upper extremities.

Associations between WMH and motor deficits of lower extremities have consistently been reported in previous studies (Baezner et al., 2008; Rosano et al., 2010; Smith et al., 2015; Kim et al., 2016; Rosario et al., 2016; Pinter et al., 2017). We found that WMH was a significant predictor of lower extremity dysfunction in a Chinese community population. The Leukoaraiosis and Disability (LADIS) Study, which was carried out in 639 non-disabled elderly individuals (aged 65–84 years), found that deficiencies in gait and balance performance characterized by stand, chair-stand, and walking tests were correlated with the severity of WM changes (Baezner et al., 2008). In the Prospective Urban Rural Epidemiological (PURE) study with 803 community participants, decreased performance in Timed Up and Go test consisting chair-stand and walking tasks was associated with higher volume of WMH (Smith et al., 2015). In our current study, motor assessment of the lower extremities included tests of gait speed and repeat chair-stand, and the correlation between WMH and gait speed did not survive the FDR corrections, which is inconsistent with a prior study, the Lothian Birth Cohort 1936 (LBC1936) study (Pinter et al., 2017). A potential explanation is that, participants in LBC1936 were on average 15 years elder than those in our study, and therefore severer WMH and greater variation of walking speed were found. This diversity might lead to greater significant differences in statistical analysis.

Similar to lower extremity functions and postural control, upper-extremity functions also serve as predictors of disability and mortality, but have been under-investigated until now (Ostwald et al., 1989; Gale et al., 2007). Importantly, we simultaneously evaluated motor function in the lower and upper extremities in this large cohort study. Interestingly, we found that WMH contributed to motor deficits in pronation–supination rather than finger-tapping, and that brain atrophy contributed to motor deficits in both pronation–supination and finger-tapping, although the correlation between brain atrophy and finger-tapping did not survive the FDR correction. Consistent with our results, the Cardiovascular Health Study (CHS) found no association between performance of finger-tapping and worsening of WM measures, although univariate analysis was used in that study and not many details were presented (Longstreth et al., 2005). Our study further extended the above investigation.

In our study, both WMH and brain atrophy were found to modulate motor function of upper extremities. As compared to that of the lower extremities, neural control of the upper extremities is a sophisticated and advanced ability, and heavily relies on integration of the visual, somatosensory, and action system of the motor cortex, and its subcortical connectivity with brainstem and cerebellum (Takakusaki, 2017). Motor performance of pronation–supination involves hand movements with visuospatial and coordination components, strength, and speed, may require the overall function of the intact nervous system (Scherder et al., 2008). Therefore, repeated tasks of pronation–supination may be sensitive to disturbances of WM integrity and cerebral degenerations.

In the present study, CSVD imaging burdens of lacunes, CMBs, and PVS were not found to be modifiers for motor parameters of the lower or upper extremities. This may be explained by the lower prevalence of lacunes in community populations than in individuals with clinically symptomatic CSVD, in addition to the presence of minimal brain structural damage of vascular origins, as assessed using imaging markers of CMBs or PVS, in community-dwelling populations.

The strengths of our current study include the relatively large sample size, the use of high-resolution MRI, improved evaluation of imaging markers of CSVD with higher accuracy, and the quantitative evaluation of motor performances speed in both the lower and upper extremities. Our study has some limitations. Gait speed was measured starting with the command “go.” Therefore, the acceleration time was included in our timing. This may explain the slower walking speed in our cohort when compared to those in other studies. Comorbid conditions such as dementia were not excluded. This may have led to either overestimates or underestimates of the associations between imaging markers of CSVD and the risk of motor impairment. However, cognitive status and educational level were adjusted for in the multivariable models, minimizing the potential bias caused by differences in cognition. Finally, this study was achieved in a rural population in China, which involved a great proportion of low-educated farmers, limiting the generalizability of our findings.

## Conclusion

Our study confirms the major impact of WMH on motor performance of both lower and upper extremities in community-dwelling individuals. We also found that brain atrophy might drive the deterioration of upper extremity functions.

## Ethics Statement

This study was carried out in accordance with the recommendations of the Medical Review Ethics Committee of Peking Union Medical College Hospital with written informed consent from all subjects. All subjects gave written informed consent in accordance with the Declaration of Helsinki. The protocol was approved by the Medical Review Ethics Committee of Peking Union Medical College Hospital (reference number: B-160).

## Author Contributions

NS drafted the manuscript and conducted the statistical analyses. F-FZ managed the database and provided additional statistical expertise related to the analysis. Y-CZ, L-YC, L-XZ, JN, MY, M-LL, Z-YJ, and S-YZ contributed to the conception and design of the study and interpretation of the data. G-LG provided expertise on brain imaging analysis. FT was the technical expert for motor performance acquisition. All authors provided final approval for the version of the manuscript submitted for publication, and agree to be accountable for the work. Y-CZ was the principal investigator of the study and was responsible for the study conception and interpretation of data, and had final responsibility for the decision to submit for publication.

## Conflict of Interest Statement

The authors declare that the research was conducted in the absence of any commercial or financial relationships that could be construed as a potential conflict of interest.
