# Supplementary material for: Cerebral Small Vessel Disease Burden Is Associated with Motor Performance of Lower and Upper Extremities in Community-Dwelling Populations
Source: Front Aging Neurosci. 2017 Sep 27;9:313. doi: 10.3389/fnagi.2017.00313 (PMC5623680; doi:10.3389/fnagi.2017.00313)
Supplement: Supplementary file 1 [file Data_Sheet_1.DOCX]

Supplementary Material

Cerebral small vessel disease burden is associated with motor performance of lower and upper extremities in community-dwelling populations

Ning Su, Fei-Fei Zhai, Li-Xin Zhou, Jun Ni, Ming Yao, Ming-Li Li, Zheng-Yu Jin, Gao-Lang Gong, Shu-Yang Zhang, Li-Ying Cui, Feng Tian, Yi-Cheng Zhu

*** Correspondence:** Yi-Cheng Zhu: zhuych910@163.com

# Supplementary Method

**Assessment of motor functions**

For motor evaluation of gait speed, participants were asked to walk at usual pace over a three-meter distance. The average time in seconds, converted into m/s, of two trials was used in the analysis. For motor evaluation of chair-stands, a subset of the Short Physical Performance Battery (SPPB), participants were asked to keep their arms folded across their chest, followed by stand up and sit down as quickly as possible five times without stopping.(Guralnik et al., 1994) For motor evaluation of pronation-supination, a subset of Scale for the Assessment and Rating of Ataxia (SARA), participants were asked to perform 10 cycles of repetitive alternation of pronation and supinations of the hand on his/her thigh as fast and as precise as possible using the dominant and non-dominant hand, respectively.(Schmitz-Hubsch et al., 2006) The time for pronation-supination of each participant was defined as the mean of both sides. For motor evaluation of finger-tapping, a technique from the Unified Parkinson’s Disease Rating Scale (UPDRS), participants were asked to tap the index finger on the thumb tip as quickly and as big as possible ten times using the dominant and non-dominant hand, respectively.(Goetz et al., 2008) The time for finger-tapping of each participant was defined as the mean of both sides.

# Supplementary Tables and Figures

## Supplementary Tables

**Supplementary Table 1. Univariate analysis of motor performance by burdens of CSVD.**

| Motor Performance |  | Total WMH | | |  | Lacunes | | |  | CMBs | | |  | PVS-WM | | |  | PVS-BG | | |
| --- | --- | --- | --- | --- | --- | --- | --- | --- | --- | --- | --- | --- | --- | --- | --- | --- | --- | --- | --- | --- |
|  |  | Mild | Severe | *p* |  | No | Presence | *p* |  | No | Presence | *p* |  | Mild | Severe | *p* |  | Mild | Severe | *p* |
| Gait speed, m/s |  | 0.915 (0.283) | 0.806 (0.174) | <0.001^†^ |  | 0.903 (0.278) | 0.831 (0.187) | <0.001^†^ |  | 0.894 (0.273) | 0.875 (0.222) | 0.547 |  | 0.896 (0.279) | 0.873 (0.204) | 0.597 |  | 0.899 (0.277) | 0.851 (0.203) | <0.05^*^ |
| Chair-stand, s |  | 8.659 (1.805) | 9.946 (2.796) | <0.001^†^ |  | 8.794 (1.991) | 9.657 (2.580) | <0.05^*^ |  | 8.848 (2.056) | 9.603 (2.468) | <0.05^*^ |  | 8.896 (2.163) | 9.059 (1.832) | 0.086 |  | 8.904 (2.126) | 9.032 (2.040) | 0.343 |
| Pronation-supination, s |  | 7.328 (1.551) | 8.284 (1.802) | <0.001^†^ |  | 7.459 (1.635) | 7.895 (1.698) | <0.05^*^ |  | 7.470 (1.614) | 8.016 (1.887) | <0.05^*^ |  | 7.519 (1.672) | 7.554 (1.551) | 0.523 |  | 7.482 (1.658) | 7.776 (1.600) | <0.05^*^ |
| Finger-tapping, s |  | 5.278 (1.641) | 5.865 (2.078) | <0.001^†^ |  | 5.337 (1.695) | 5.750 (2.032) | 0.055 |  | 5.347 (1.692) | 5.866 (2.195) | 0.086 |  | 5.398 (1.789) | 5.403 (1.583) | 0.601 |  | 5.377 (1.767) | 5.529 (1.694) | 0.179 |

Abbreviations: WMH = white matter hyperintensities; CMBs = microbleeds; PVS-WM = dilated perivascular spaces in white matter; PVS-BG = dilated perivascular spaces in basal ganglia.

^†^ *p* <0.001.

## ^*^ *p* <0.05

## Supplementary Figures

**Supplementary Figure 1.**


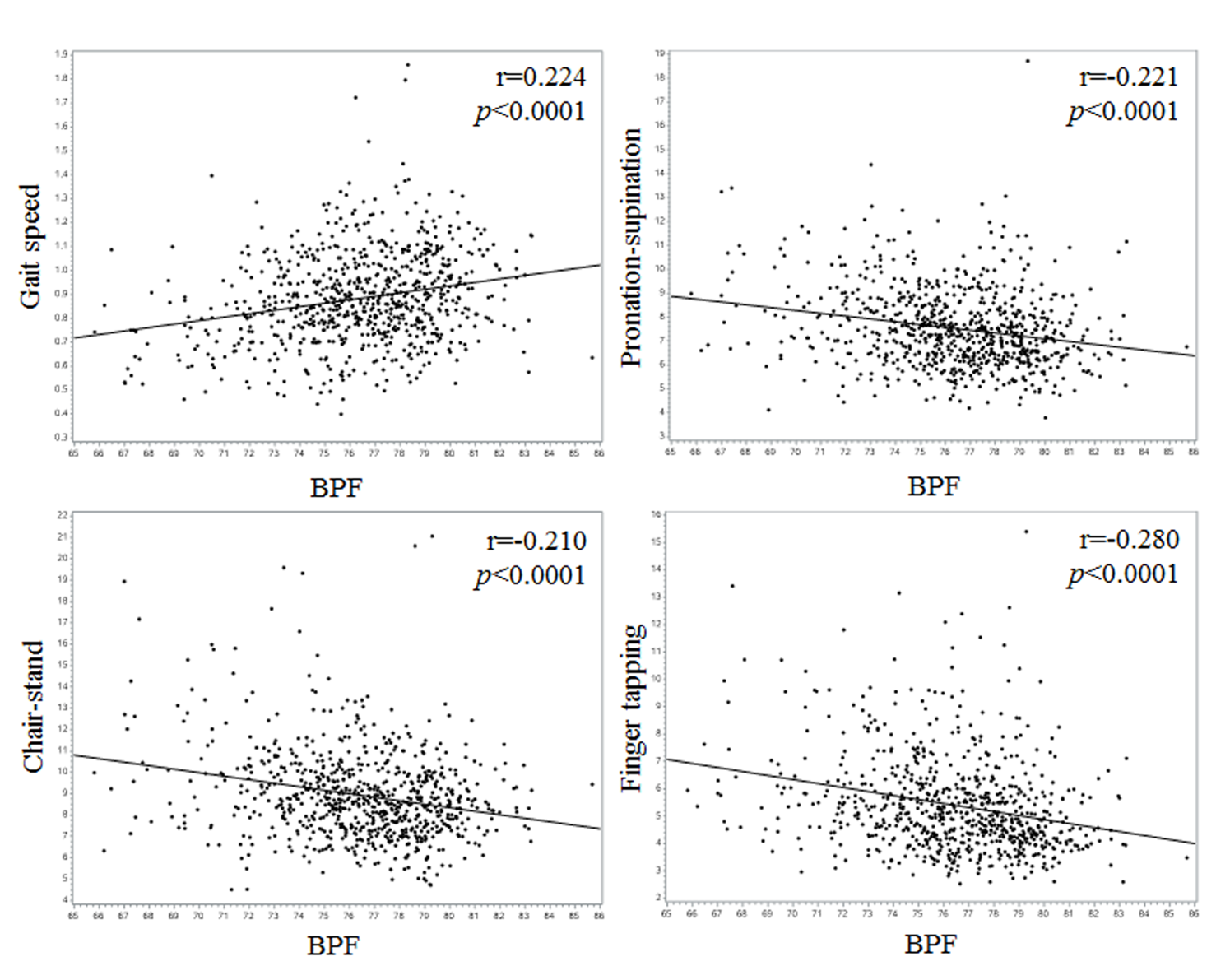


**Supplementary Figure 1.** Correlation between motor performance and brain atrophy in community population. Spearman’s correlation analysis was applied to assess the correlation of motor performance of lower extremities (gait speed, time of chair-stand) and upper extremities (time of pronation-supination, time of finger-tapping) with BPF, a marker of brain atrophy. Abbreviations: BPF = brain parenchymal fraction.

**Supplemental References:**

Goetz, C.G., Tilley, B.C., Shaftman, S.R., Stebbins, G.T., Fahn, S., Martinez-Martin, P., et al. (2008). Movement Disorder Society-sponsored revision of the Unified Parkinson's Disease Rating Scale (MDS-UPDRS): scale presentation and clinimetric testing results. *Mov Disord* 23(15)**,** 2129-2170. doi: 10.1002/mds.22340.

Guralnik, J.M., Simonsick, E.M., Ferrucci, L., Glynn, R.J., Berkman, L.F., Blazer, D.G., et al. (1994). A short physical performance battery assessing lower extremity function: association with self-reported disability and prediction of mortality and nursing home admission. *J Gerontol* 49(2)**,** M85-94.

Schmitz-Hubsch, T., du Montcel, S.T., Baliko, L., Berciano, J., Boesch, S., Depondt, C., et al. (2006). Scale for the assessment and rating of ataxia: development of a new clinical scale. *Neurology* 66(11)**,** 1717-1720. doi: 10.1212/01.wnl.0000219042.60538.92.
